# Expanding Understanding of Motherhood Penalty: How Gaps in Family Policies Contribute to Gaps in Old-Age Earnings in Russia

**DOI:** 10.3389/fsoc.2019.00067

**Published:** 2019-09-20

**Authors:** Marina A. Kingsbury

**Affiliations:** Department of Political Science, University of Alabama in Huntsville, Huntsville, AL, United States

**Keywords:** inequality, family policy, Russia, motherhood penalty, gender

## Abstract

This study identifies policy shortcomings in the structure of Russian family policies and old-age pension calculations and shows how gaps in coverage contribute to the motherhood penalty in Russia. I first show that shortages in access to affordable childcare contribute to mothers' involuntary labor market inactivity leading to loss in earnings. I then discuss how childcare breaks are treated in terms of pensionable experience and present simulation of pension outcomes to show the negative impact of long childcare breaks on mothers' pensions.

## Introduction

Russian family policies, inherited from the communist past, extend maternity, parental leave coverage, and affordable childcare to families with children. Women continue to actively participate in labor force in modern Russia, despite the collapse of communist planned economy, that encouraged female labor participation by sharing childcare responsibilities (Pascall and Manning, 2000; Ogloblin, 2005; Razzu, 2016). Post-communist labor market experiences brought former non-market economies on par with the such socio-economic consequences as the gender wage gap (Waldfogel, 1997; Budig and England, 2001; Correll et al., 2007; Miller, 2011; England et al., 2016). Although studies report a general trend of closing the gender wage gaps (Waldfogel, 1998; Blau and Kahn, 2003, 2016), this trend does not universally describe the experiences of all employed women. Parenthood dampens mothers' positions compared to fathers or childless women who are making advances in closing the gender gap (England, 2005; Correll et al., 2007; Misra and Strader, 2013).

Mothers, unlike childless women, experience a persistent wage gap. Women provide most care for children, a collective good valued by societies (England and Folbre, 1999; Lewis and Giullari, 2005). Despite societal expectations of high-quality care, mothers pay a disproportionally high price, motherhood penalty, manifested in diminished income caused by occupational segregation, reduced employment, discrimination at hiring, and promotion connected to perceptions of incompetence and lack of commitment, and loss of tenure, skills, and other components of human capital during childcare breaks (Budig and England, 2001; Mandel and Semyonov, 2005; Correll et al., 2007; Budig et al., 2012; Boeckmann et al., 2015; Cools and Strøm, 2016; Javornik, 2016; Roosalu and Hofacker, 2016).

Public policy mechanisms can mitigate the negative effects of career breaks on mothers' wages (Budig et al., 2010; Fodor and Kispeter, 2014; Boeckmann et al., 2015; Cukrowska-Torzewska, 2017). While scholars have extensively focused on the levels of compensation, length, and eligibility requirements for maternity, and parental leave and childcare policies, less attention has been paid to how these policies complement each other making the transition from one level of protection to another seamless. Using the relatively understudied case of the Russian Federation, I show that the gaps in coverage of the transition period from paid parental leave to state-provided childcare exacerbate the motherhood penalty in both the short-term and long-term. Public childcare in Russia is primarily provided by municipal childcare facilities regulated by federal and local laws that cover educational, sanitary, and nutritional aspects of care. In the short term, lack of childcare options for youngest children push women out of workforce because there is an 18 month gap in family policy coverage between the expiry of paid parental leave and onset of state-provided childcare guarantee.

In the long term, lack of childcare for the youngest children prolongs childcare breaks from employment leading to lower social insurance retirement income. Currently the application of pension credits for parenthood fail to mitigate the motherhood penalty on old-age earnings. This finding contributes to the existing scholarship by stressing the connection between the design of family policies and mothers' old-age earnings. To quantify this relationship, I report results of pension benefits simulation for women with and without children to show that motherhood has a long-term effect on future pensions. The use of the novel simulation methodology allows the assessment of the existing policy effects on mothers' earnings. The simulation is validated by testing its results on a representative sample of Russian mothers who reported taking parental leaves. The paper contributes to the extant research by presenting a framework that expands the institutional discourse to highlight its implications for the old-age income. I show that family policies can have long-lasting effects that amplify gender inequalities in the old age.

## Theoretical Setting

### Gender Wage Discrimination

This study investigates the effects of family policies on mothers' earnings and seeks to disentangle how the continuity of family policies affect mothers' wage-earning ability and eligibility for old-age pensions. The paragraphs below review the current academic discussion on gender wage discrimination in the short and long-term and discuss whether the existing debate applies to the post-communist context. Wage discrimination has been identified as a most profound tangible manifestation of motherhood penalty (Budig and England, 2001; Miller, 2011; England et al., 2016). Mothers with smallest children experience the largest motherhood penalty (Harkness and Waldfogel, 2003). Motherhood wage penalty may stem from discrimination based on perceptions of incompetence or lack of commitment. Correll et al. (2007) find that mothers in the US are expected to poorly perform due to lack of focus and commitment, thus are scrutinized more heavily leading to such quantifiable losses as offers of lower starting salaries. Benard and Correll (2010) show that competent professional mothers are discriminated in performance evaluations by being viewed as less warm and less likable. Glass and Fodor (2011) report that employers in Hungary channel mothers into lower-paid positions because of perceived lack of devotion to the job. Hungarian employers, especially in higher-paid business and finance sectors, screen out mothers during the hiring process and deny return to workplace after parental leave despite legal protections (Glass and Fodor, 2011, 2018). Other studies show that motherhood contributes to productivity penalty leading to stymied professional advancement and career growth (Wolfinger et al., 2008; Krapf et al., 2017). Hook and Pettit (2016) find that highly-educated women are less likely to become mothers or to have more than one child to avoid gender wage segregation and meager career advancement.

Career breaks due to childrearing exacerbate motherhood wage penalty in the short-term (Albrecht et al., 1999; Harkness and Waldfogel, 2003). Employment patterns of mothers differ from men and childless women. Mothers take career breaks to provide care while men do not, except for mandatory paternity leave breaks such as one instituted in Sweden. Budig and England (2001) report a 7% motherhood wage penalty per child, of which about one-third is caused by childcare-related breaks. Miller (2011) estimates that for each year of postponement of motherhood, women's earnings can increase by 9%. Erosa et al. (2016) find that women suffer a gap in human capital accumulation because of having children, which translates in a 40% increase in gender wage gap for women between ages of 20 and 40 when return on human capital is high. Budig et al. (2016) estimate that mothers lose about 15% of their annual earnings per child.

In the long term, old-age inequality is a result of penalties that accumulate over women's life (Meyer and Herd, 2007; Kahn et al., 2014). Gender pension gaps form when women are engaged in low-paid employment, part-time employment, or take breaks from employment (Ginn and Arber, 1993; Meyer and Bridgen, 2008; Leitner, 2011; D'Addio, 2013; Grady, 2015; Rutledge et al., 2017). Women's pensions are connected to lower lifetime earnings due to occupational segregation into lower-earnings' sectors, discrimination at hiring or promotion of women, and lower pensionable experience due to career breaks associated with parenthood responsibilities (Ginn and Arber, 1993; Hakim, 2005; Meyer and Bridgen, 2008; Meyer et al., 2013; Hagewisch and Hartmann, 2014; OECD, 2015; Hook and Pettit, 2016; Herd et al., 2018).

### Motherhood Penalty: Institutional Approach

A large body of research connects motherhood wage penalty to institutions of the welfare state. Welfare states provide healthcare, childcare and education, public pensions, taxes and tax credits, parental leave, disability, unemployment, and other well-being benefits. Welfare state institutions differ in the ways they conceptualize eligibility in connection to labor market participation, financing sources, and breadth of coverage based on need (Esping-Andersen, 1990). These differences shape societal outcomes such as class relations, income inequality, access to social insurance, and shape gender inequality structures.

Gender scholars have expanded the welfare state regime literature by arguing that the original classification is based on a male breadwinner experience and needs while ignoring the experiences of women in the contemporary workforce (Orloff, 1993, 2009; Lewis, 1997; Lewis et al., 2008; Esping-Andersen, 2009; Saxonberg, 2013; Hobson, 2018). The gender approach to the welfare state centers on whether the state balances, supports, or ignores the needs of women in performing the caregiving function (Fraser, 1994; Korpi, 2000; Gornick and Meyers, 2003, 2008). Attention is drawn to the design of the welfare state policies such as maternity and parental leave as well as childcare policies that accommodate the variety of paths taken by modern mothers. On the one side of the spectrum, there are the conservative welfare states that focus on caregiving by maintaining long paid leaves, family allowances, supporting flexible working hours, and part-time work. This approach reinforces traditional gender roles of a male breadwinner and woman caregiver that is connected to greater chances of old-age poverty (Misra et al., 2007b). The opposite side of the spectrum are the dual earner-career approach, often exemplified by the Scandinavian welfare state model. It not only supports mother's labor force attachment through generous family policies but also challenge traditional gender roles through policies that encourage or mandate shared caregiving by both parents. The liberal welfare state prioritizes women's labor force participation and market-based delivery of care services. Thus, welfare state regimes foster different patterns of family policy institutions, that have become an important determinant of women's experiences on the labor market (Korpi, 2000). The emphasis on elements of family policies as an explanatory factor of contemporary mothers' work-family relations is referred to an institutional approach to explaining gender equality, as opposed to the cultural-normative aspect of gender roles within family and at a work place (Boeckmann et al., 2015).

Design of family policies can shape women's employment by either contributing to larger wage penalty or reducing it by facilitating labor market attachment (Pettit and Hook, 2005, 2009; Misra et al., 2007a, 2011; Hegewisch and Gornick, 2011). Paid maternity leave, parental leave, and childcare support the attachment to labor market participation and thus help increase earnings, protect mothers from forced exit from the labor force, aid in maintaining mothers' work-life balance, and increase overall tenure, which helps reduce wage penalties (Harkness and Waldfogel, 2003; Lewis and Campbell, 2007; Misra et al., 2007a; Gornick and Meyers, 2008; Hegewisch and Gornick, 2011; Misra and Strader, 2013). Maternity leave is designed for caring of a newborn and commences at childbirth or, in some countries like Russia, a few weeks before childbirth. Maternity leave compensation is distinct in many countries by high levels of wage replacement. Parental leave starts after the expiry of maternity leave. Compared to maternity leave, it lasts longer than maternity leave but is compensated at lower rates or may feature an uncompensated term. Public childcare includes a range of services from government-run care centers to government-subsidized or government-regulated care centers. High-quality available and affordable childcare institutions facilitate full-time employment at affordable rates (Gornick and Meyers, 2003).

Empirical evidence finds that maternity leave reduces gender inequality and contributes to mothers' attachment to labor force. A comparative study of ten European countries found that one week of paid maternity leave reduces wage penalty by 5.3% (Hallden et al., 2016, p. 12). Budig et al. (2016) find that paid maternity leave lasting for 25 weeks reduces motherhood penalty per child by 6%. However, the relationship between parental leave and gender equality is not straightforward. While paid parental leave in general is considered beneficial for mothers, specific gains depend on the length of parental leave. Short paid parental leave lasting no more than 1 year is found to aid mother's labor force attachment (Boeckmann et al., 2015; Javornik, 2016). Budig et al. (2016) find that moderately-timed well-paid parental leave of up to 2 years lowers motherhood penalty, but the relationship between parental leave and motherhood penalty is curvilinear. Misra et al. (2011) find that moderate-length paid leave reduces chances of maternal poverty. The effects of long parental leaves, exceeding 2 years in length, are less straightforward. Motherhood penalty increases when parental leave approaches 3 years due to employer discrimination or loss of human capital (Misra et al., 2007a, p. 819; Pettit and Hook, 2009; Boeckmann et al., 2015, p. 18; Budig et al., 2016). Cukrowska-Torzewska (2017) finds that long parental leave reduces maternal employment in countries with low childcare coverage, an outcome especially prevalent in the post-communist CEE countries. Fodor and Kispeter (2014) show that long or poorly-paid parental leave promotes maternal caregiving and leads to labor force detachment as well as increases chances of maternal poverty. Rules of parental leave uptake introduce another dimension to the impact of leave policy (Leitner, 2003; Ciccia and Verloo, 2012; Javornik, 2014, 2016). States, such as Sweden, allow for sharing of care responsibilities by extending and encouraging parental leave to both parents. This can help alleviate the negative career impact of childcare on women (Javornik, 2014). This leads to the first hypothesis:

*Hypothesis 1: long parental leave should increase motherhood penalty*.

Childcare policies, especially for children under 3 years old, reduce motherhood penalty (Ronsen and Sundström, 2002; Pettit and Hook, 2005, 2009; Misra and Strader, 2013; Cukrowska-Torzewska, 2017). Care for children bears vital gendered implications. Welfare state's institutions can aid mothers with caring functions by funding or subsidizing high-quality affordable childcare, including nurseries and preschools. Availability and affordability of childcare facilities has far-reaching consequences for work-life balance, earnings history, and broader gender equality. Boeckmann et al. (2015) show that childcare diminishes gaps in mothers' employment, but motherhood penalty can reach 18% in countries that do not provide access to affordable full-time quality childcare. Borisov (2017) connects childcare breaks to shorter employment history for Russian women when compared to men. Each year of employment adds 1.6% to earnings of Russian women with university degree, thus childcare breaks lead to quantifiably lower earnings and shorter length of overall employment. Misra et al. (2007b) find that availability of childcare decreases the likelihood of female poverty with stronger effect than family benefits. Hallden et al. (2016) find that childcare mitigates possible motherhood penalty by 1.6% for each percent increase in childcare enrollment. Harkness and Waldfogel (2003) and Lewis (2009) stress the importance of quality full-time childcare for children younger than 3 years old, arguing that childcare for the youngest children is the scarcest and but vital for mothers' labor force attachment. Karabchuk and Nagernyak (2013) find that the likelihood of return to work for Russian mothers is significantly reduced if they care for children under 3 years old. Where childcare is only available part time, women are unable to return to full-time work, thus are channeled to low-paid part-time employment (Pfau-Effinger, 2005).

*Hypothesis 2: Low childcare enrollment rates for children aged 1.5–3 years old should correspond to higher motherhood penalty*.

Gendered policy implications are evident in the effects of pension designs. Career interruptions disadvantage mothers, especially in systems where pension outcomes are connected to contributions (Leitner, 2001; Ginn and MacIntyre, 2013; Grady, 2015). D'Addio (2013) finds that women, who interrupt their careers for child-rearing, suffer a 10% reduction in pension replacement rates after a 5-year break, 22% reduction after a 10-year break, and 33% reduction after a 15-year career break. Ginn (2003) occupational pensions due to occupational segregation and care responsibilities. Finch (2014) argues that in the UK mothers extend their work years beyond the retirement age to make up for lost income.

In countries with social insurance pension schemes, old-age benefits are calculated based on the number of years in labor force. Childrearing can dampen future pension benefits if caring for children lead to labor market inactivity (Grady, 2015; Borisov, 2017). Countries that guarantee public pensions can mitigate the negative effects of career breaks for childcare using policy mechanisms, such as inclusion of care periods as pensionable experience (Ginn, 2004; Vlachantoni, 2011). Pension credits represent a form of compensation for socially significant activities such as childrearing (Leitner, 2001; Herd, 2005; Vlachantoni, 2011; Herd et al., 2018). In general, pension credits for periods of inactivity, including childcaring, have been found to have a positive effect on future pensions (Ginn and MacIntyre, 2013; OECD, 2015). D'Addio (2013) finds that child-care pension credits reduce the motherhood penalty by 3 to 7%, depending on the pension scheme and the length of career breaks. Scholars, however caution against overestimating the value of compensating mothers with either means-tested mechanisms or pension credits for care functions without addressing the underlying focus on defining pensions based on male-centered full-time uninterrupted work history (Leitner, 2001; Marier, 2007). The next hypothesis reflects the gendered implications of the pension design:

*Hypothesis 3: Pension credits should decrease motherhood penalty in old age benefits by accounting for the lost income during childcare leaves*.

### The Russian Context

Much of the extant research focuses on the advanced Western democracies. This study covers a less-studied case of the Russian Federation. The Russian case provides valuable insights into the complex nature of motherhood penalty in a country with paid maternity and parental leave and public childcare, pension credits for childcare, and high female employment rates. Russian Federation inherited from a communist past a set of family policies including paid leave, childcare facilities, baby bonus programs, and family allowances (Rivkin-Fish, 2010; Avdeyeva, 2011; Chernova, 2012; Sinyavskaya, 2016). Currently, a Russian mother is entitled to a fully-paid 140-day maternity leave and a one-time baby-bonus payment of 16,350 rubles (US$266). Employed Russian mothers receive 100% wage replacement rates for the duration of maternity leave. Unemployed mothers are compensated based on the fixed rate indexed yearly (Sinyavskaya, 2016). The partially-paid parental leave commences immediately after maternity leave and lasts until the child is one-and-half years old. It is compensated at the levels of 40% of previous wage with the minimum payment set at 3,066 rubles (US$50) for the first child, and 6,131 rubles (US$100) for the second and more children. The maximum benefit for high-earning mothers is 23,089 rubles (US$426). As the child reaches 18 months, a mother can take what is widely considered an unpaid parental leave until the child is 3 years old. Additional parental leave is compensated in the form of a family allowance in the amount of 50 rubles (US$0.80), making this payment so negligible, that it is safe to refer to the extended parental leave as unpaid. Workplace guarantee is reserved for up to 3 years of parental leave. Mothers giving birth to a second or more child are entitled to the Maternity Capital Certificate, a one-time non-cash benefit that can be invested into mother's pension, child's education, or applied toward a mortgage payment for a dwelling (Avdeyeva, 2011; Sinyavskaya, 2016). Additional family benefits may be provided by regional and local administrations, but scope of support varies by location and are subject to budgetary constraints. For example, the government of Saint Petersburg pays additional family benefit per child for low income families disbursed to debit cards to be redeemed at specialized children's stores for the purchase of child-related goods like diapers, clothing, shoes, or formula. All Saint Petersburg families receive a one-time baby bonus payment at birth of a child.

In post transition years, the Russian Federation overhauled its old-age pension policies. After a botched attempt at a three-pillar pension scheme, in 2015 the government introduced a pension formula that structures the social insurance retirement based on earned pension coefficients (Eich et al., 2012). Recognition of life experiences during economically active years is an important part of the reformed pension scheme. The assignment of pension coefficients for childrearing is an attempt to increase its societal value, a part of the larger demographic strategy championed by President Putin. Initially, the idea of including the entire period spent caring for children, irrespective of the number of children, was introduced by the Vice-Premier for Social Policy Olga Golodets (RG, 2012). If implemented in full, this policy initiative had a chance to positively affect the problems of work-life balance in Russia by recognizing care work without placing term limits. However, fiscal conservatives in the government intervened by proposing thresholds on the duration of care work that could be considered pensionable experience. Maxim Topilin, then Head of the Labor Ministry, supported the idea that childcare work should be recognized, but his Ministry's policy proposal limited the total compensated childcare period to the maximum of 4.5 years (Malykhin, 2013). The new pension law adopted the conservative policy proposed by the Labor Ministry in 2014. It instituted per-child limits of 1.5 years with a maximum ceiling that was extended to 6 years by 2015.

The Russian case presents an interesting dynamic between the structure of the family policies and old-age pensions given that Russia remains one of the last developed states with the lowest female retirement age, still at 55 years. The literature on post-communist Central and Eastern Europe (CEE) fits the region in the broader family policy context of developed democracies (Gal and Kligman, 2000; Glass and Fodor, 2007, 2011; Szelewa and Polakowski, 2008; Fodor and Kispeter, 2014; Javornik, 2014; Blum, 2016; Razzu, 2016; Roosalu and Hofacker, 2016; Cukrowska-Torzewska, 2017; Fodor and Glass, 2018). In the past, socialist governments pursued full employment of mothers by funding paid maternity and parental leave, government-subsidized childcare and subsidies for families with children (Rudd, 2000; Haney, 2002; Cook, 2007; Rivkin-Fish, 2010). High female employment was driven by shortage of labor in closed planned economies and declining fertility, thus states shared the caregiving burden with women as a part of the socialist social contract (Einhorn, 1993; Pascall and Manning, 2000, p. 248; Fodor et al., 2002; Haney, 2002; Szikra and Tomka, 2009). In Russia, the transition to market economy shifted the policy accents on care obligations. The state no longer aimed to share childcare obligations in pursuit of full employment. Childcare delivery, care options, and responsibility was shifted to parents, overwhelmingly women, who nonetheless continued to work at high rates. The communist social contract was replaced with a new one centered on an autonomous family which makes own life decisions including employment and childcare. The primacy of market mechanisms in defining policy needs and services introduced a greater variety of care options but emphasized the parental autonomy over caregiving choices (Chernova, 2013).

Post-socialist transition set CEE countries on divergent paths (Fodor and Glass, 2018). Privatization, free-market demands, and shrinking public and service sector jobs channeled women out of labor market (Razzu, 2016). The end to the near-full compulsory employment patterns of the past contributed to the widening of gender wage gap and spiking female poverty (Kligman, 1994; Fodor, 2002; Fodor and Horn, 2015). The change manifested in acute problems with reconciling work and care, high unemployment and job insecurity, along with the devaluation of mothering and care. Saxonberg and Sirovátka (2006) describe the profound push in re-introducing traditional gender roles, or “re-familialization,” manifested in repudiation of practices associated with the communist rule, specifically the dual-earner family structure, and the renewed emphasis on cultural and religious views on family roles practiced before the Soviet rule took over (Fodor et al., 2002; Szelewa and Polakowski, 2008; Inglot et al., 2012). Russia, along with the rest of the CEE countries underwent transformation from the dual-earner near-full employment socialist heritage to employment driven by demands of market economy (Pascall and Manning, 2000; Lewis, 2001; Cerami, 2006; Pascall and Kwak, 2009). In Russia, soaring unemployment was compounded by the disruption of childcare provision when enterprises were no longer obligated by the state to provide social welfare services to its workers.

Extant research estimates that the wage gap due to motherhood in post-communist Central and Eastern Europe differs by country (Razzu, 2016). In Russia, gender wage gap persists around one-third of a man's salary (Glinskaya and Mroz, 2000; Kalugina et al., 2009). Newell and Reilly (1996) report that in 1990–1995 the gender wage gap in Russia was about 30%, attributing the gap to gender differences. Ogloblin (2005) calculates that a long-run gender wage gap in Russia is close to 31%. Atencio and Posadas (2015) report that the adjusted gap in hourly wages has fluctuated around 28% since 1994. Labor market structure adds to the persistence of the Russian gender wage gap, with greater gender wage gap reported in male-dominated occupations (Glinskaya and Mroz, 2000). Klimova (2012) finds significant female occupation segregation leading to over-representation of Russian women in low-skilled low-paying jobs. Gerry et al. (2004) find that while the wage gap remains stable after an initial post-soviet collapse increase, it disproportionately targets low-income female workers.

In sum, this paper expands on the institutional approach to explaining causes and mitigating effects of motherhood penalty in the context of the post-socialist family policy setting (Blum, 2016). The literature belabors the impact of various configurations of separate leave policies on maternal employment and the size of motherhood penalty. I draw attention to the importance of a seamless transition from paid parental leave to high-quality affordable childcare and connect the gaps in childcare coverage to motherhood penalty in wages and pensions.

## Data and Methodology

To illustrate the impact of motherhood on earnings, I first discuss the scope of family policy coverage in Russia, focusing on the limits of state-run childcare coverage. I present enrollment data to show that childcare is primarily provided by public institutions, detail shortage of available slots, and use female employment data to elaborate on the support the hypothesized connection between long parental leaves, lack of childcare, and motherhood penalty in earnings. The results are based on the data and author's calculations derived from the yearly statistics and published survey data (Savinskaya, 2011; Rosstat, 2017a).

What follows is the discussion of pension outcomes for Russian women who took childcare breaks from employment. First, I simulate pension outcomes for an average Russian mother factoring in a variation of common childcare leave periods using a web-based pension calculation tool, and second, I test the simulation's outcomes on the sample of Russian mothers. This approach is a form of a static simulation usually employed to estimate the impact of public policy on citizens (Mitton et al., 2000).

**Figure 1:**
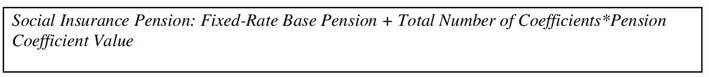
Russian social insurance pension formula.

The Pension Fund of the Russian Federation (PFRF) makes available a *pension calculator*, an interactive tool that helps the Russian citizens to estimate the future social insurance pension benefits. The calculator factors in work history, including childcare breaks, and earnings. Currently, social insurance pensions are comprised of the fixed-rate base benefit, established, and indexed by the government, and insurance pension, determined by the number of pension coefficients as expressed in monetary terms (Figure 1). Pension coefficients are accrued yearly if an employed individual makes social insurance contributions of 16% of her wage. Monetary value of individual contributions is divided by the pre-determined maximum social insurance payment to find the total yearly number of accrued pension coefficients. The maximum number of coefficients that can be accumulated in 2017 was 8.26. The value of pension coefficients is indexed yearly by the government. Thus, social insurance pension benefits are largely a function of length of employment and wages.

For the simulation, I calculate future pensions for hypothetical scenarios of earning histories for women with children, holding age, and wages constant while allowing for education, care breaks, and number of children to vary. Childless women serve as a base reference point that is compared against pension outcomes of mothers with one, two, or three children who take parental leave of 18, 28, or 36 months. For women without a university degree the overall employment history without career breaks is set at 37 and 33 years for women with a university degree. Female retirement is 55 years. Levels of education are modeled via salary values and length of employment. To focus on the effects of motherhood on pension outcomes, I set the assumptions of no other career breaks in women's employment history, except for childcare. Wage value for college-educated women adopted at 42,000 rubles (US$685), corresponding to an average wage of a public-sector employee. Wages for women without a college degree is adopted at 28,000 rubles (US$457), an average salary of a retail cashier (Rosstat, 2017b). The ratio of salaries based on education assumes a premium on college education of 33%, a premium that fits the reported range between 30.4 and 42.8% depending on occupation (Belokonnaya et al., 2007). The Pension Calculator sets all other intervening variables such as workplace discrimination, lack of opportunities, or income inequality at constant. These factors undoubtedly affect women's labor market experienced when women are discriminated at hiring, retention, and promotion. However, the purpose of this simulation is to model the effects of labor market inactivity that is not covered as a pensionable experience. Detailed data sources are discussed in the Data Appendix.

To test the simulation's assumptions, I predict pension outcomes for a subset of mothers based on the representative Russian household survey data of 60,000 households (Rosstat, 2017a). The survey contains responses from mothers who completed parental leave in 2016 (*N* = 719). These data do not include wage information but provide average family income that includes all family earnings. To separate mothers' salaries from men's, I limit the inquiry to a subsample of single mothers with children (*N* = 145). To calculate the penalty in pension benefits related to childcare breaks, I estimate the total length of employment based on age, reported actual past work history, reported number of children and duration of parental leave assuming no other career interruptions until the onset of retirement. Motherhood penalty is estimated as a ratio of earnings lost due to childcare career breaks as compared to single mothers in the subsample who took no childcare leave.

## Findings

### Russian Childcare Provision: Gaps That Contribute to Motherhood Penalty

Despite comprehensive paid maternity and parental leave family policies, the abrupt ending of state support after 18 months produces a gap in coverage. There is no income replacement for the period of unpaid parental leave (ages 28 to 36 months) and the government-provided childcare obligation does not onset until the age of 36 months. Childcare facilities for younger children exist, but attendance rates are low due to severe shortage of available slots. Thus, Russian mothers who are unable to secure childcare before or at the end of paid parental leave face the increased prospects of incurring motherhood penalty until the childcare coverage becomes available (Hypothesis 2). I support this argument by examining data on childcare enrollment, childcare availability, and motherhood employment.

**Figure 2:**
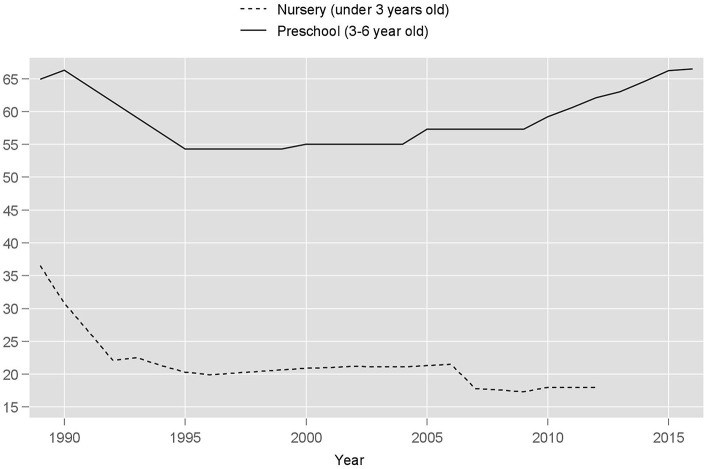
Public childcare enrollment percentage in Russia*. Source, Rosstat and UNICEF; *UNICEF nursery data are available only through 2012.

The total number of state-run preschools has declined 2-fold between 1990 and 2012 despite the growing number of children, leading to shortage of available slots observed by the year 2010 (Table 1). In 2012, the government pledged to increase financing of existing childcare infrastructure and building new facilities to care for children ages three and older (Savitskaya, 2004; President of RF, 2012). Enrollment of older children returned to the pre-collapse level by 2014, while attendance of nurseries declined from 31% in 1990 to 18% in 2012 (Figure 2). In 2016, only 12.8% of Russian household survey respondents with youngest children indicated satisfied need for public childcare. At the same time, 49% of families stated they needed nursery care, but it was unavailable (Rosstat, 2017a). The emphasis on the age of three as a coverage threshold cemented the gap in family policy coverage, leaving the needs of younger children unmet.

**Table 1:** Government preschools in Russia 1990–2016.

**Year**	**1990**	**1995**	**2000**	**2005**	**2010**	**2011**	**2012**	**2013**	**2014**	**2015**	**2016**
Preschools, (thousands)	87.9	68.6	51.3	46.5	45.1	44.9	44.3	43.2	51.0^*^	50.1	49.4
Enrollment, (thousands)	9,009	5,584	4,263	4,530	5,388	5,661	5,983	6,347	6,814	7,160	7,343
Children per 100 slots	108	83	81	95	107	106	105	105	106	106	105

Source: Rosstat (2014; 2017c);

* *Including facilities not functioning or under renovation*.

The end of paid parental leave is a pivotal point in mother's life course. Russian scholars define three patterns of return to work for mothers with small children: first, at expiry of paid maternity leave, second, at the end of paid parental leave, and, third, at the end of unpaid parental leave. Savinskaya (2011) reports that an equal share of Moscow mothers, 18% each, exercised the first two scenarios, while 30% remained on parental leave for 3 years. In 2016, mean duration of parental leave for women who re-entered workforce was 2.3 years, indicating that on average Russian mothers remain out of workforce 9.6 months longer than covered by paid parental leave (Rosstat, 2017a). The chief reason for the long gaps in employment of Russian mothers is the lack of accessible government childcare.

**Figure 3:**
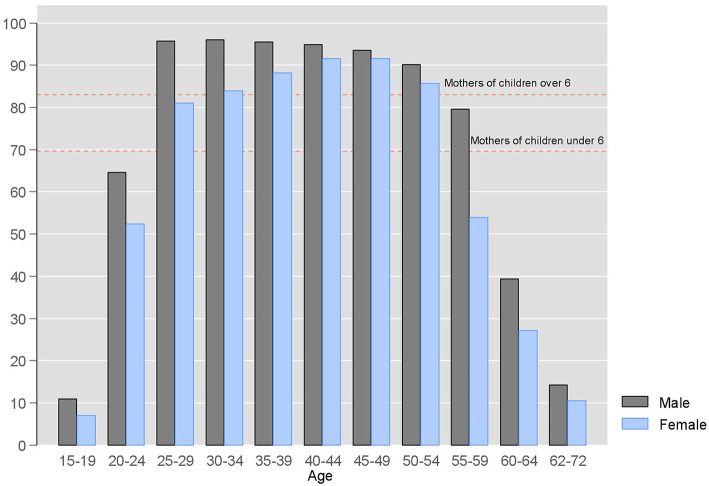
Labor force participation percentage in Russia by sex and age in 2016. Source: (Rosstat, 2017b).

Despite impediments, Russian women continue to highly value work. Employment data show a 9% increase of labor market participation for women older than 35 when compared to 25 to 29 age group, the average age for the first childbirth (Figure 3). Women with preschool age children are 13% less active in the workforce than mothers of older children. Participation rates of mothers of older children outpace childless female employment rate (81%), indicating unwavering preference of Russian mothers to returning to paid employment.

Strategies for dealing with the shortage of accessible childcare vary. Those who have relatives nearby rely on unpaid care by other female family members. Although, in a departure from past practices, modern Russian grandmothers remain employed longer and are less available or unwilling to assume unpaid full-time care for grandchildren (Zdravomyslova, 2014). Private childcare facilities are unaffordable for most families. In Moscow, prices can reach up to 25 times higher than government preschools (Forbes.ru., 2012). Nation-wide, costs of private childcare exceed that of government-run preschools 6-fold (HSE, 2016). The share of children attending private facilities remain in the 1.2–2% range around the country (Rosstat, 2017c). Engagement of private nanny services is reported by 4–6% of families (FOM, 2004; Savinskaya, 2011).

These data support the argument that the main source of childcare in Russia remains government-run preschools and childcare facilities. The lack of available government childcare and prohibitive cost of private facilities force women to remain unemployed, limiting their choice of life and career paths. Thus, the flaw in family policy coverage leaves women unsupported for the duration of unpaid parental leave, manifesting in motherhood penalty. As hypothesized, gaps in public childcare availability for children under 3 years old, hampers mothers' ability to return to work leading to penalty in earnings. The next section describes the way the state treats childcare breaks for calculation of pension benefits and connects childcare breaks to motherhood penalty in pension earnings.

### Pension Earnings and Motherhood Penalty: Simulation Results

Under the Russian social insurance pension schema, pension coefficients assign values to various life experiences during the economically active years. Care for children older than 1.5 years of age receives no recognition in pension calculations. The distribution of pension coefficients benefits women who care for a second, third, and fourth children. One year spent caring for the first child yields 1.8 pension coefficients, but 1 year spent caring for the second child yields 3.6 coefficients, and 1 year spent caring for a third and fourth children yields 5.4 coefficients. Reportedly, one-third of Russian mothers care for their children for 3 years. Many women report that long parental leave breaks are forced because affordable government childcare is unavailable. The 2015 pension formula only covers half of this period in determining pension benefits. Thus, long career breaks impose motherhood penalty in pension benefits.

To illustrate the negative impact of career breaks on pension earnings, I first simulate pension outcomes in a typical scenario of a life course based on the pension formula spelled out in Figure 1. Table 2 shows the difference in estimated pension benefits depending on the number of children, employment history, and length of parental leave. The calculations are performed for women born in the year 1979, who are active on labor market and are done having children. Choosing 1979 as the base year sets the woman's age to thirty-nine, the age at which most are done with childbearing. However, the formula calculation applies to all women with birth year 1967 and later.

**Table 2:** Simulated pension outcomes for Russian women.

**Scenario**	**Number of children**	**Years of parental leave**	**Years in workforce**	**Total pension coefficients**	**Pension value, rubles**	**Comparison to base pension, %**	**Motherhood pension penalty, %**
**PENSION OUTCOMES FOR WOMEN WITH COLLEGE EDUCATION**							
1	0	0	33	189.00	19,656	100	0
2	1	1.5	31.5	181.06	19,048	96	4
3	2	3	30	180.70	19,004	96	4
4	3	4.5	28.5	177.30	18,737	95	5
5	1	2.3	30.7	177.50	18,755	95	5
6	2	4.6	28.4	168.50	18,045	92	8
7	3	9.2	26.1	161.90	17,526	89	11
8	1	3	30	175.00	18,556	94	6
9	2	6	27	163.44	17,648	90	10
10	3	9	24	154.28	16,929	86	14
**PENSION OUTCOMES FOR WOMEN WITH SECONDARY EDUCATION**							
11	0	0	37	141.95	15,959	100	0
12	1	1.5	35.5	137.00	15,571	98	2
13	2	3	34	138.51	15,689	98	2
14	3	4.5	32.5	158.12	15,723	99	1
15	1	2.3	34.7	134.00	15,334	96	4
16	2	4.6	32.4	129.40	14,971	94	6
17	3	9.2	30.1	127.15	14,797	94	6
18	1	3	34	133.11	15,265	96	4
19	2	6	31	127.00	14,785	93	7
20	3	9	28	123.60	14,517	91	9

*Based on the 2017 rate of 78.58 rubles per pension coefficient and base social insurance pension of 4,805 rubles (PRF, 2017). Age at first birth is 25, age at second birth is 29.5, and age at third birth is 32 years old (Human Fertility Database). The age of labor force entry is assumed at 18 and 22 for secondary and college education respectively. Wages for college-educated are set at 42,000 rubles and for secondary-educated at 28,000 rubles. Base pensions are benefits calculated for women who do not interrupt employment for childrearing*.

Life scenarios 1–10 predict pension outcomes for a woman with a college degree. Scenarios 1 and 11 are the base scenario for college-educated and non-college-educated childless women, respectively. Women without a college degree have a longer work history, due to starting employment 4 years prior to college graduates, but earn less over the course of their lives due to lower wages. Scenarios 2–4 and 12–14 calculate pension benefits for mothers who only withdraw from employment to uptake paid parental leave. Scenarios 5–7 and 15–17 present outcomes based on the reported average leave duration in Russia for the last available survey year (Rosstat, 2017a). Scenarios 8–10 and 18–20 show pension outcomes for women who take long unpaid parental leave. The simulation illustrates the comparative disadvantage in pension outcomes women face when taking childcare breaks, not the precise amount in rubles that a pensioner would collect.

Holding salary and age constant, I show that mothers incur a reduction in future pension benefits, as compared with benefits of childless women. The size of motherhood pension penalty, calculated as a ratio of mothers' pensions to pensions of childless women (base scenario), varies by length of a childcare break. Those mothers who manage to return to the workforce after the end of paid parental leave are at an advantage to those who take long unpaid parental leave. For example, compared to childless women with post-secondary education and the same earnings, educated mothers with one child incur a 4% penalty for taking paid parental leave, 5% penalty for additional 9.6 months of unpaid parental leave, and 6% penalty when staying on unpaid parental leave for 18 months until the child's third birthday. Pension penalty remains the same for college-educated mothers with two children taking paid parental leave only. Taking paid parental leave with three children leads to a 5% pension penalty.

**Figure 4:**
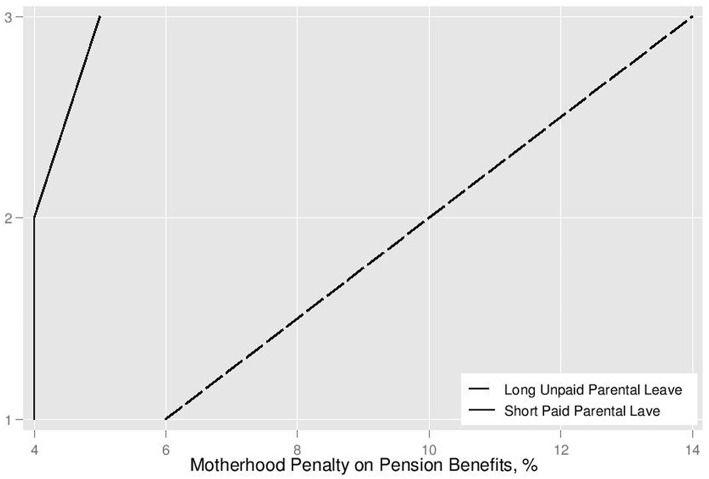
Pension penalty for childcare breaks for college-educated mothers, by number of children.

Overall, the simulation results validate Hypothesis 1 that long and unpaid parental leave has a greater impact on future pension benefits. The penalty grows substantially with second and third children. Figure 4 plots pension penalties for college-educated mothers by length of parental leave. These data are the ratio of mother's pensions to the base pension of childless women; they can be found in the last column of Table 2. They indicate that even with pension credits, childcare breaks diminish mothers' pensions, but the number of children, employment history, and length of childcare breaks influence the total size of the pension gap. The difference in pension penalties by number of children is smaller for women with secondary education who have longer work history which off-set the career breaks. Higher earners with shorter careers incur a greater penalty for unpaid parental leave that expands with each child, as evident from the plot of penalty for college-educated mothers.

Pension penalty is the smallest for all mothers sticking to paid parental leave only, but non-college educated women with three children are expected to see their pension outcomes improved reflecting the higher coefficient value for the third child. This simulation suggests that the intended design of the pension formula to reward women with more than one child may have a positive impact only for a limited subset of mothers who take short paid leave, care for three children, and have long employment histories (Scenarios 12–14). Pension penalty for mothers with secondary education halts its expansion for mothers of two children.

The results of the simulation in Table 2 indicate that motherhood penalty on pensions is connected to childcare breaks and rises with the number of children and length of childcare breaks. I test these results using a subset of single mothers from the Russian Comprehensive Monitoring of Living Conditions (RCML) 2016 data (Rosstat, 2017a). RCML is an internationally-recognized reliable comprehensive representative survey of the Russian households that includes data on incomes, family structures, health, and well-being. RCML includes data on the number and ages of children in the household, length of parental leave uptake, and met and unmet need for childcare enrollment. The use of RCML data on actual parental leave uptake and childcare enrollment is used here to validate the simulation results, which are constructed based on the assumptions about predicted characteristics of Russian mothers. RCML data reveals that on average, single mothers took 2.2 years of parental leave. A part of the sample, 38% of mothers, have not interrupted their employment for parental leave. These results are not surprising given that in the absence of a second wage earner mothers seek to minimize breaks in employment. Of those who did take parental leave, 73% incurred unpaid leave ranging from 1 to 31 months on top of the paid 8-month leave. Ten women reported remaining on unpaid parental leave over 3 years. On average, women in this sample reported 13 years of schooling, indicating some combination of a secondary and vocational training. Average sample wages are 32,740 rubles (US$518).

The employment history of mothers who took parental leave is 27.8 years. It is on average 4.8 years shorter than employment history of mothers without career interruptions (*t* = 6.2; *p* = 0.00). The average overall motherhood penalty for this sample of single mothers who took unpaid parental leave is 6%. These statistics confirm that childcare breaks significantly reduce employment histories of Russian mothers, leading to lower pension earnings.

**Figure 5:**
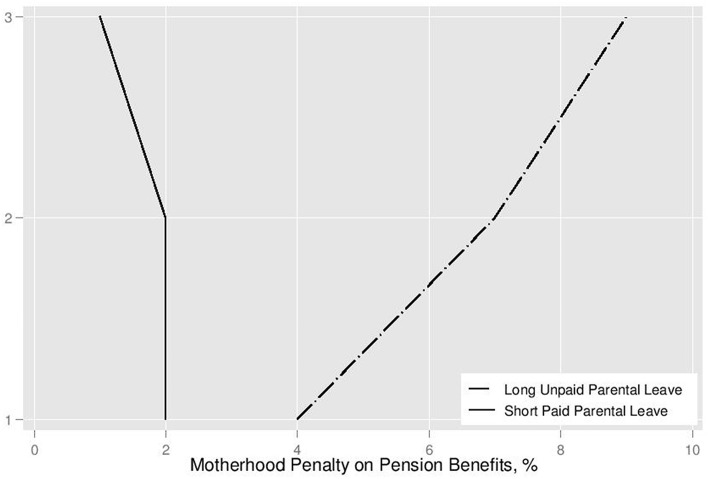
Pension penalty for childcare breaks for non-college-educated mothers, by number of children.

Figure 5 details average motherhood penalty by duration of parental leave and the number of children. These data confirm the simulation findings presented in Table 2 in that they show that motherhood penalty grows with each child and differs by the duration of childcare breaks. The Mann-Whitney test indicate that there is a statistically-significant difference between motherhood penalty incurred during paid (average 0.64; S.D. 1.35) and unpaid (average 5.99; S.D. 3.33) parental leave (*t* = −12.74; *p* = 0.00). This evidence lends support to Hypothesis 1.

As the average education years reported in the sample are below what is expected for a college degree (at least 14), I compare these results to Scenarios 11–20 of the Table 2. Scenarios 12–13 predict that the motherhood penalty for paid parental leave should be 2% for each of the first two children. These results are supported by the microsimulation outcomes spelled out in Figure 5. The motherhood penalty dropped, however insignificantly, for the third child, somewhat contradicting Scenario 14 predictions, which expect a whole percentage point reduction in penalty. As expected, unpaid parental leave imposes a motherhood penalty in pension benefits that grows with each additional child. Motherhood penalty on pensions incurred for unpaid parental leave for one child is 4.7%. It increases to 6.9% for the second child, reducing future pension by additional 2.2 percentage points. Motherhood penalty for unpaid leave for the third child is the greatest at 7.6%, reducing pension benefits by an additional 0.7 percentage points. This means that motherhood penalty on social insurance pension in Russia grows by 47% for the second child, and by 10% for the third child. These findings render mixed support for the Hypothesis 3, which postulates that pension credits for decrease motherhood penalty. Limited nature of pension credits that cover paid parental leave negate the potential egalitarian feature of this policy mechanism.

## Discussion

Women approach greater equality when they are less constrained in life choices be it employment, fertility, motherhood, or family status. Studies show that short paid leave policies and accessible childcare help mothers to reconcile caring duties with careers. Paid leave alleviates the negative impact of career breaks, while accessible childcare provides women choice of strategies and timing for combining motherhood and labor market activity (Lewis and Giullari, 2005; Gornick and Meyers, 2008; Lewis, 2008; Hobson, 2011; Cukrowska-Torzewska, 2017).

This paper contributes to the institutional literature, by arguing that, in addition to examining the levels of compensation and length of coverage, it is necessary to consider how individual policies complement each other creating continuous coverage to parents. I show that when the state provides paid parental leave and workplace guarantees but allows for gaps in coverage between paid leave expiry and guaranteed childcare provision, mothers incur motherhood penalty. The structure of Russian family policy creates a serious impediment to mothers wishing to return to gainful employment at the end of paid parental leave because the state-provided childcare is scarce but private childcare options are unaffordable.

This research contributes to the existing debate on motherhood penalty by providing contextual evidence for the argument that long and poorly-paid parental leave has a negative effect on maternal employment (Budig et al., 2012; Hallden et al., 2016). I qualify this argument by specifying that when long and essentially unpaid parental leave remains the only option of affordable care, it amplifies motherhood penalty. Thus, it is not only the structure of parental leave but its relationship to the lack of affordable childcare options that contribute to motherhood penalty.

Further, I show that the combined impact of unpaid parental leave and lack of childcare for the youngest children increase motherhood penalty in a complex way: it not only leads to career interruptions and loss in wages, but also affects pension benefits in the long-term. It is important to consider how family policy design impacts retirement income in a country with social insurance pension scheme. When the state limits pension benefits to periods of paid parental leave, mothers incur a long-term motherhood penalty in pensions due to labor market inactivity during unpaid parental leave. Lack of government childcare pushes Russian mothers into taking unpaid parental leave causing not only loss of wages but also pension benefits. I calculate, based on a subsample of single Russian mothers, that unpaid parental leave reduces pension benefits by 6% with variations based on the number of children.

The way the Russian pension policy is designed signals the strong preference by the state to encourage women to re-enter workforce after the end of paid parental leave of 18 months. If women are unable to combine care and work, they are penalized because additional parental leave is not included into the pensionable experience and thus reduces future pensions. Moreover, as my simulation shows and the analysis of survey data confirms, pension credits do not alleviate the pension penalty. Women taking paid parental leave still incur pension penalty.

This penalty applies not only to women who want to re-enter workforce but cannot secure childcare. It also affects women who wish to continue to care for their children at home and choose to not re-enter workforce. Through the design of pension formula, the state explicitly favors working mothers who re-enter workforce at the end of paid parental leave, limiting life and career choices. Experiences of all other mothers is neglected by not being included into pensionable experience.

An important shortcoming and long-term impact of Russian family policy is its potential contribution to female poverty. Poverty among women in old-age is a significant concern due to lower lifetime wages, career breaks, and female longevity. Pension credits for parental leave can mitigate future old-age female poverty. In Russia, however, the existing pension formula does not eliminate the gender gap in pension allocation. Women do not receive full credit for all years spent caring for their children. Women who have only one child are disadvantaged more than women who have two or three children. These pension credit rules decrease the overall value of future pensions, contributing to the heightened probability of old-age poverty among women. The low retirement age threshold compounds the severity of motherhood penalty in Russia. Russian retirement age remains the lowest across Europe at 55 years for women, despite longer female life expectancy.

## Conclusion

Despite the provision of paid parental leave and government-financed childcare, Russian family policy lacks a comprehensive approach to address the main drivers of motherhood penalty: gender gap due to career breaks for childrearing. This creates systematic disadvantages for Russian mothers who struggle to combine work and family obligations, especially when their children are of preschool age. Given that access to public childcare in Russia is neither guaranteed nor plentiful, mothers remain chief caregivers sans real choice.

To connect the gaps in family policy coverage to long-term effects of childcare breaks on mothers' retirement earnings, I simulated pension outcomes for hypothetical scenarios and tested the simulation results using survey data to show that mothers' pensions are reduced because of childcare breaks. The pension credits cover childcare breaks only partially and do not eliminate the penalty entirely. The loss of pension is incurred mainly during unpaid childcare leave. In effect, the state does not support the entire range of life choices for mothers by not committing to providing care to children under the age of three. These family policy limitations appear to negate the potential gains of paid maternity and moderately-timed paid parental leave on motherhood penalty in Russia and instead incur a greater motherhood penalty that extends into the old age.

This study has implications for further research. On the design of family policy in connection to motherhood penalty, this research calls for further systematic focus on the continuity of coverage by family policies and the connection of family policy design to pension benefits. Given that there is a clear connection between family policy and pension outcomes, what would be the best policy design that diminishes the long-term motherhood penalty? Russian female retirement age is one of the lowest in Europe. Should the Russian government increase the retirement age and would the increase in retirement age alone alleviate motherhood penalty on pension benefits?

## Author Contributions

This work was an original research completed by MK.

### Conflict of Interest Statement

The author declares that the research was conducted in the absence of any commercial or financial relationships that could be construed as a potential conflict of interest.
