# Supplementary material for: Expanding Understanding of Motherhood Penalty: How Gaps in Family Policies Contribute to Gaps in Old-Age Earnings in Russia
Source: Front Sociol. 2019 Sep 20;4:67. doi: 10.3389/fsoc.2019.00067 (PMC8022753; doi:10.3389/fsoc.2019.00067)
Supplement: Supplementary file 1 [file Data_Sheet_1.docx]

**Data Appendix**

Data for this paper are collected from the official government statistic on preschool attendance. The maternity, parental leave, family allowance benefits, and pension benefits are compiled from the government agencies’ reports. Preschool attendance data have been compiled from yearly publications by the Russian Federal State Statistic Service (Rosstat) and Higher School of Economics (HSE). Rosstat makes available data on the total number of preschools and the number of enrolled children. Shortage of preschools is calculated based on the numbers reported by the annual Comprehensive Monitoring of Living Conditions survey (Rosstat, 2017a). The data on the existing maternity, parental benefits, and child allowances are drawn from the yearly statistical updates published by the Russian Social Insurance Fund, the leading Russian legal database Consultant Plus, and regional Social Insurance administrators. To cross-validate data, I consulted the Russian Federation country summary available from the International Review of Leave Policies and Research 2016 (Sinyavskaya, 2016). The main data collection effort was undertaken in 2017.

Analysis of the pension benefits relies on data that were gathered through analyzing the provisions of the Russian Pension Code as well as analytic memos published by the Russian Pension Fund, the government agency in charge of administering the pension program and distributing pension benefits (Eich, Gust, and Soto, 2012; PFRF, 2017). Pension reform information was gathered through the content analysis of speeches by the Russian cabinet members in charge of pension administration (see below). Wage data are taken from Rosstat. Average age of birth for first, second, and third child are taken from the Human Fertility Database.

| **Pension Legislation** | | | |
| --- | --- | --- | --- |
| Federal Law N162-FZ | Changes to pension Legislation | http://www.pfrf.ru/info/order/organization_appointment_payme/~3969 |  |
| Federal Law N 400-FZ | “On Insurance Pensions” | https://rg.ru/2013/12/31/strahpensii-dok.html |  |
| Federal Law N166-FZ | “On State Pension Provision in the Russian Federation” | http://www.consultant.ru/document/cons_doc_LAW_34419/ |  |
| Federal LawN422-FZ | “On Guaranteeing the Rights of Insured Persons in the Social Security Pension System of the Russian Federation” | https://rg.ru/2013/12/30/pens-dok.html |  |
| Federal Law N197-FZ | Labor Code of the Russian Federation December (30, 2001) | http://www.consultant.ru/document/cons_doc_LAW_34683/ |  |
| Pension Calculator | Pension Fund of the Russian Federation | http://www.pfrf.ru/eservices/calc/ |  |
| Calculation of pensions | Pension Fund of the Russian Federation | http://www.pfrf.ru/grazdanam/pensions/kak_form_bud_pens/ |  |
| Calculation of pensions | On applicability of pension credits for childcare for grandmothers | http://www.pfrf.ru/branches/kuzbass/news~2014/09/30/41883 |  |
| Calculation of pensions | On including periods of childcare as employment history | http://www.pfrf.ru/branches/moscow/news~2019/02/19/177453 |  |
| Calculation of pensions | On applying pension credits for childcare as per the 2015 pension formula | http://www.pfrf.ru/branches/tver/news~2017/09/13/142395 |  |
| Calculation of pensions | On applying pension credits for childcare review of past legislation | http://www.bashinform.ru/likbez/920175/ |  |
| Calculation on pensions | On minimum social insurance pensions | http://www.pfrf.ru/grazdanam/pensionres/soc_doplata/ |  |
| **Russian socio-economic data** | | |  |
| Wages data | Rosstat  “Труд и Занятость в России 2017б” Table 01-03 | http://www.gks.ru/free_doc/doc_2017/trud_2017.pdf |  |
| Education Data | Higher School of Economics | https://www.hse.ru/primarydata/oc2016  https://www.hse.ru/primarydata/orf2014 |  |
| Fertility | Human Fertility Database | https://www.humanfertility.org/cgi-bin/countrypage.php?country=RUS |  |
| Employment by age | Rosstat “Труд и Занятость в России 2017” | http://www.gks.ru/free_doc/doc_2017/trud_2017.pdf |  |
| **Preschool data** | | |  |
| Preschool Enrollment | Rosstat form #85-K | http://www.gks.ru/free_doc/new_site/population/obraz/doshkol-obr.htm |  |
| Preschool enrollment | Rosstat “Россия в Цифрах 2014” | http://www.gks.ru/free_doc/doc_2014/rus14.pdf |  |
| Preschool enrollment | Rosstat “Россия в Цифрах 2017” | www.gks.ru/free_doc/doc_2017/rusfig/rus17.  pdf |  |
| Preschool enrollment | Rosstat “Семья, Материнство и Детство” | http://www.gks.ru/wps/wcm/connect/rosstat_main/rosstat/ru/statistics/population/motherhood/ |  |
| **Family Policy coverage and benefits** | | |  |
| Parental leave and childcare need | Rosstat Comprehensive monitoring of living conditions 2016 | http://www.gks.ru/free_doc/new_site/KOUZ16/index.html |  |
| Parental Benefits in Saint-Petersburg | Government of Saint-Petersburg | https://www.gov.spb.ru/gov/otrasl/trud/denezhnye-vyplaty-v-sankt-peterburge-razlichnym-kategoriyam-grazhdan-v/ |  |
| Parental Benefits | Consultant Plus legal database | http://www.consultant.ru/law/ref/poleznye-sovety/detskie-posobija/posobie-po-uhodu-za-rebenkom/ |  |
| Parental Benefits | Federal Social Insurance Fund | https://portal.fss.ru/fss/sicklist/child15-guest |  |
| Parental Benefits | Sinyavskaya (2016) | https://www.leavenetwork.org/fileadmin/Leavenetwork/Annual_reviews/2016_Full_draft_20_July.pdf |  |
| Parental Benefits | Government of Moscow | https://www.mos.ru/otvet-semya-i-deti/list-rozhdenie-rebenka-kak-oformit-vse-dokumenty-i-poluchit-denezhnye-vyplaty/ |  |
| Childcare coverage | Rosstat (2012) “Как живут дети в России” | www.gks.ru/publish/prezent/infograf/children_russia.pdf |  |
| Childcare coverage | OECD Family Database | ht www.oecd.org/els/family/database.htm |  |
| **Russian officials about pension credits** | | |  |
| Olga Golodets | Interview to Rossiyskaya Gazeta | www.rg.ru/2012/12/05/denznak.html |  |
| Interfax | Interfax about pension credits | http://www.interfax.ru/business/279203 |  |
| Aleksei Kudrin | “Russia’s Former Finance Minister Kudrin Urges Decisive Pension Reform.” | Ht https://www.reuters.com/article/russia-pensions-kudrin/russias-former-finance-minister-kudrin-urges-decisive-pension-reform-idUSL5N16P552. |  |
| Vladimir Putin | “Путин Ответил На Вопрос О Повышении Пенсионного Возраста.” | https://rg.ru/2017/11/29/putin-otvetil-na-vopros-o-povyshenii-pensionnogo-vozrasta.html. |  |
